# CATH: expanding the horizons of structure-based functional annotations for genome sequences

**DOI:** 10.1093/nar/gky1097

**Published:** 2018-11-06

**Authors:** Ian Sillitoe, Natalie Dawson, Tony E Lewis, Sayoni Das, Jonathan G Lees, Paul Ashford, Adeyelu Tolulope, Harry M Scholes, Ilya Senatorov, Andra Bujan, Fatima Ceballos Rodriguez-Conde, Benjamin Dowling, Janet Thornton, Christine A Orengo

**Affiliations:** 1 Structural and Molecular Biology, University College London WC1E 6BT, UK; 2 European Bioinformatics Institute, Wellcome Trust Genome Campus, Hinxton, Cambridgeshire CB10 1SD, UK

## Abstract

This article provides an update of the latest data and developments within the CATH protein structure classification database (http://www.cathdb.info). The resource provides two levels of release: CATH-B, a daily snapshot of the latest structural domain boundaries and superfamily assignments, and CATH+, which adds layers of derived data, such as predicted sequence domains, functional annotations and functional clustering (known as Functional Families or FunFams). The most recent CATH+ release (version 4.2) provides a huge update in the coverage of structural data. This release increases the number of fully- classified domains by over 40% (from 308 999 to 434 857 structural domains), corresponding to an almost two- fold increase in sequence data (from 53 million to over 95 million predicted domains) organised into 6119 superfamilies. The coverage of high-resolution, protein PDB chains that contain at least one assigned CATH domain is now 90.2% (increased from 82.3% in the previous release). A number of highly requested features have also been implemented in our web pages: allowing the user to view an alignment between their query sequence and a representative FunFam structure and providing tools that make it easier to view the full structural context (multi-domain architecture) of domains and chains.

## INTRODUCTION

CATH is an online, publicly accessible resource that provides a classification of protein domains based on structural data deposited into the worldwide Protein Data Bank (wwPDB) (1). The resource splits 3D structures into constituent domains (considered as semi-independently folding globular units) and clusters these domains into homologous superfamilies where there is sufficient evidence of evolutionary similarity (2). CATH uses a number of sensitive structure-comparison and sequence comparison tools (including SSAP (3), HMMER3 hmmer.org, PRC (4)) to assist the manual curation of these remote evolutionary relationships. Homologous superfamilies are organised into a hierarchy of Class, Architecture and Topology according to an increasing degree of structural similarity.

Knowledge of 3D protein structure is hugely valuable when investigating remote evolutionary relationships and detailed functional mechanisms, however the number of solved 3D structures is relatively small compared to the number of protein sequences. In order to harness the information available in the sequence databases, CATH uses a set of representative structural domains to ‘seed’ a set of sequence alignments (HMMER, hmmer.org). These alignments are converted into hidden Markov models (HMMs) and the resulting library of sequence-based ‘fingerprints’ is used to identify the location of closely related domains within protein sequences taken from the genome databases UniProtKB (5) and Ensembl (6).

By combining protein structure and sequence, the CATH resource provides comprehensive structure-based domain superfamily assignments to over 95 million protein sequences (7). In many cases, these annotated proteins are already associated with a considerable amount of additional functional annotation (e.g. UniProtKB provides annotations such Gene Ontology (GO) (8), Enzyme Commission (EC) terms (9) and Catalytic Site Atlas (10)).

The Homologous Superfamily (H) level is useful for investigating distant evolutionary relationships as it groups domains that have the same structural core but may have structural embellishments to that core that result in diverse functions. Therefore, to investigate more finely tuned features, such as the evolution of functional sites, superfamilies are also broken into smaller, more focussed groups of domains called Functional Families (FunFams). FunFams aim to group domain sequences that perform a similar function and are created via a functional classification protocol based on distinguishing sequence patterns (11,12). The resulting clusters of domains have been found to be more functionally coherent than other domain-based resources (11). While this is a non-trivial task (13–16), it is extremely useful when attempting to correlate functional annotations with sequence patterns (11). Function prediction pipelines based on the FunFams have been consistently ranked among the top 10 function prediction methods by the international CAFA competition (17,18). Since structures within FunFams tend to be highly conserved (even at low sequence identity), these clusters have also been found to be useful when suggesting templates for 3D modelling (19).

The resource allows users to search for matching FunFams by submitting a query protein sequence to the web pages (cathdb.info/search/by_sequence), or through the API (20). Finding a significant match to a FunFam can provide specific functional information (eg by mapping highly conserved positions onto the query sequence) and provide more general annotations such as most likely GO term annotations.

In 2017, CATH was named as a Core Data Resource (CDR) within ELIXIR, one of only 18 CDRs across Europe (and one of only nine outside of the EBI) (21).

## CATH v4.2 RELEASE HIGHLIGHTS

**Figure 1.**
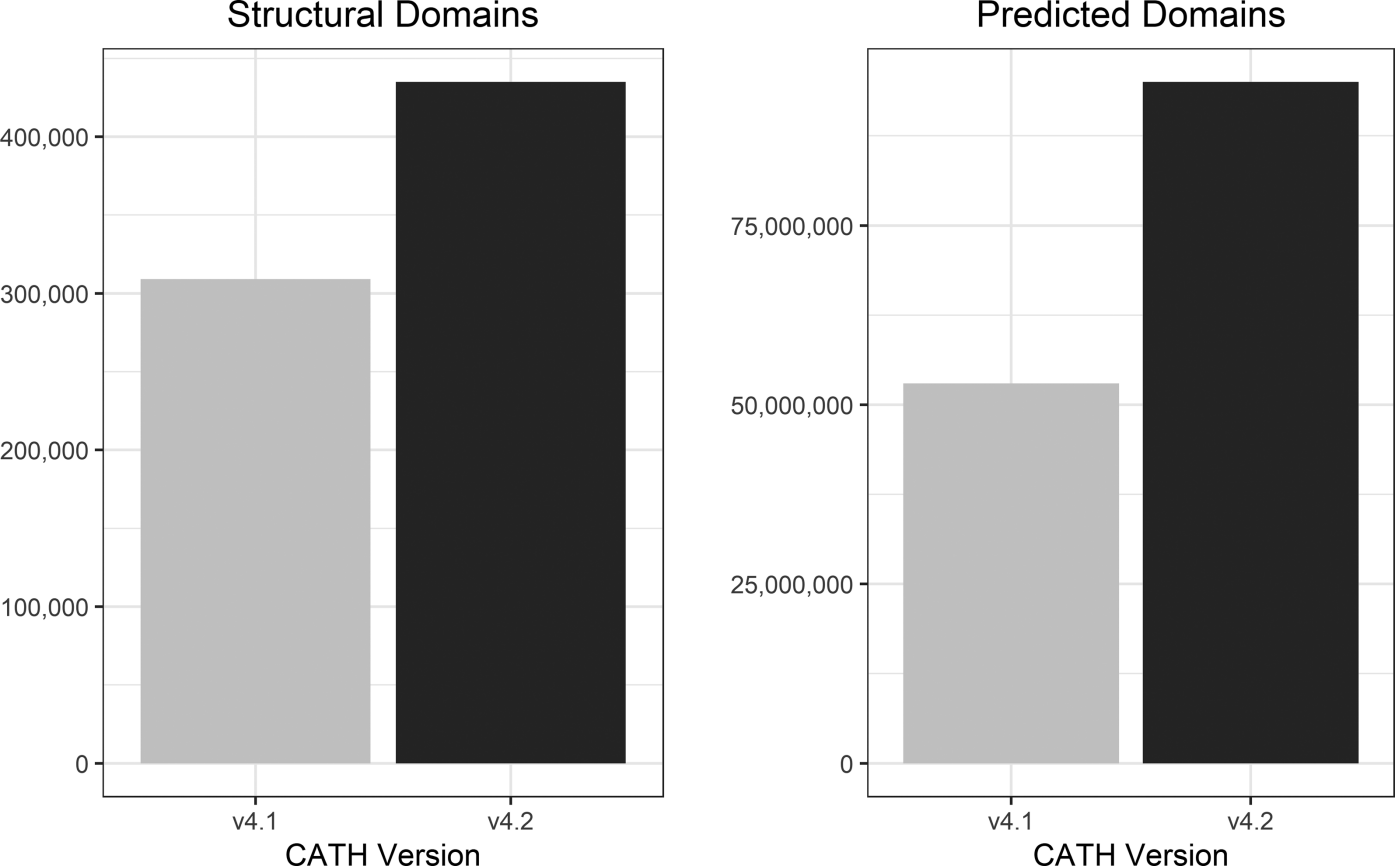
Comparison of the structural domains and predicted (sequence) domains between CATH+ releases 4.1 and 4.2.

The most recent CATH+ release, version 4.2 (based on PDB as of July 2017), brings a huge number of annotations (125 858 newly classified domain from 24 765 newly processed protein structures from the wwPDB); an increase of 40% since CATH+ release 4.1 (based on PDB as of January 2015) (Figure 1). This was made possible through the implementation of a machine learning algorithm that allows annotations to be automatically inherited when sequence and structure comparisons to existing CATH domains met a carefully benchmarked set of criteria (22). Extensive manual curation enabled the recognition of new folds, and new superfamilies within known folds.

The corresponding sequence data for this release added an extra 45 million predicted domain sequences. Fitting these additional domain sequences into the alignments for each FunFam has increased the average information content in these alignments by 13% (where information content is calculated by DOPS score, a measure of the global similarity of sequences within an alignment (23)).

**Figure 2.**
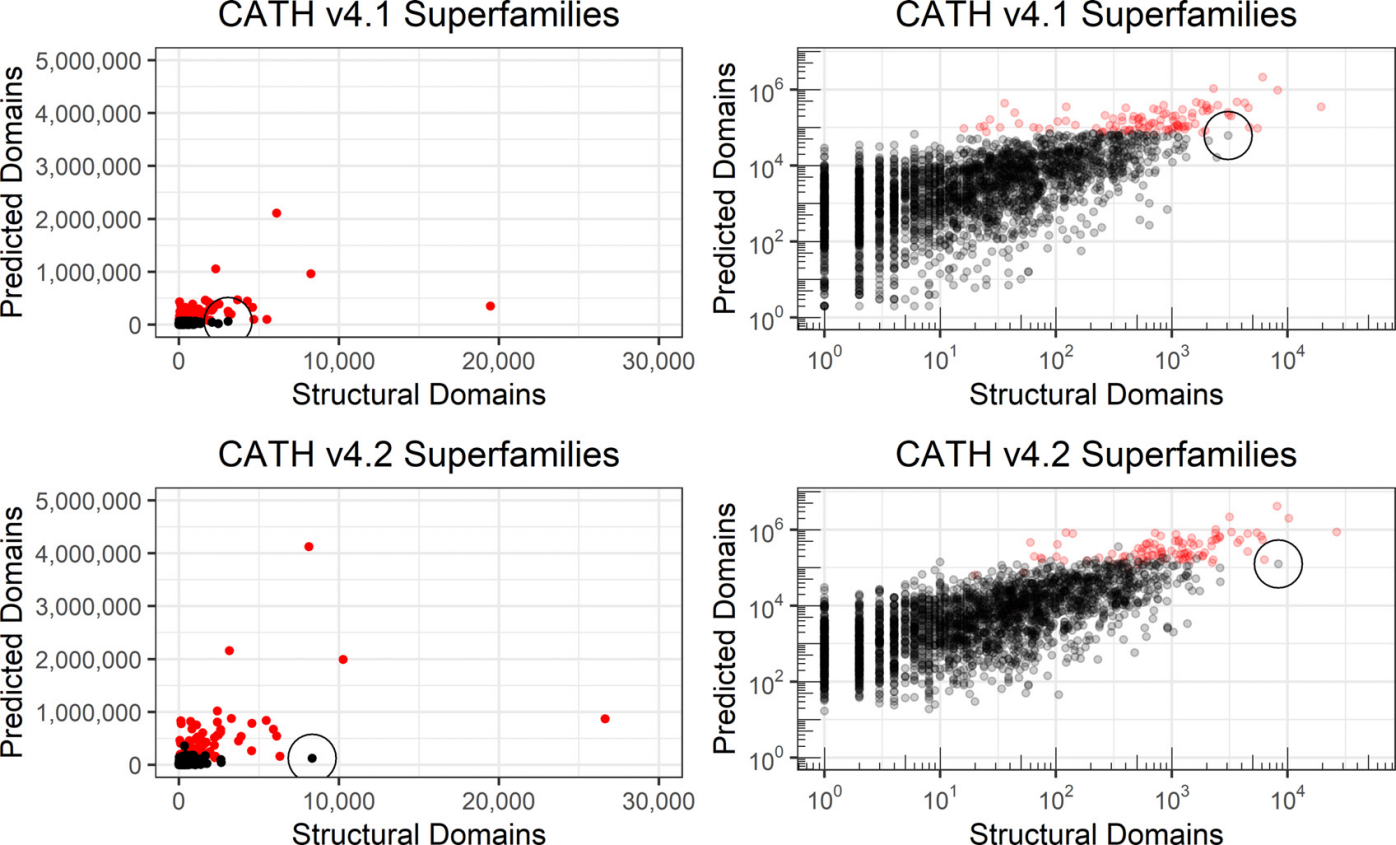
Superfamilies in CATH v4.1 and v4.2 highlighting the number of structural domains and predicted domains (shown with a linear and logarithmic scale). Each dot represents a superfamily and the largest 100 superfamilies (according to number of predicted domains in CATH v4.1) have been highlighted in red. These superfamilies contain more than half of all known protein domains. To help illustrate the growth of data, an example superfamily (3.60.20.10) has been circled in each plot. The number of structural domains in this superfamily increased 2.7-fold from v4.1 to v4.2 (3074 to 8328 domains), however this corresponded to only a small increase in the number of predicted domains. On investigation, this superfamily contains domains from a number of large proteasomes, which contain many copies of identical (or very similar) structural domains.

This release also sees the number of superfamilies in CATH rise from 2737 (version 4.1) to 6119 (version 4.2) (Figure 2), though this only corresponds to a relatively small number of new folds (18) and one new architecture. Almost two-thirds of these new superfamilies (62%) correspond to superfamilies that contain only one representative structural domain (non-redundant at 95% sequence identity). In these cases, it is often possible to detect structural and sequence similarities to existing superfamilies however the evidence does not yet reach the required threshold for these new members to be merged. It is important to note that this evidence is being updated constantly as new sequences, structures and functional annotations are added to public databases so these superfamilies may be merged in future releases. As in previous releases, we offer strictly non-redundant datasets of CATH domains in which it is guaranteed that no two domains have more than 20%/40% sequence identity (at 60% overlap). If groups are interested in using CATH domains for benchmarking or training their own algorithms, it is strongly recommended that they take advantage of these datasets (see Availability).

CATH+ adds many layers of information on top of the classification of domain boundaries and Homologous superfamily, and the process of creating a CATH+ release takes approximately 12–18 months. The first step in the process of generating a CATH+ release is to ‘freeze’ the incoming data from the wwPDB. While this is important to ensure consistency when calculating additional layers of information, it also means that the latest PDB structures may be missing from a CATH+ release. To address this, we offer a daily snapshot containing the very latest domain boundary and superfamily annotations (CATH-B). At the time of writing (Sep, 2018), CATH-B contained a further 46 906 structural domains since CATH+ 4.2 was ‘frozen’ (July 2017).

## ADDITIONAL FEATURES IN CATH

### Improved superfamily names and descriptions

Since the last release, there has been considerable effort to review existing superfamily names and provide names for unnamed superfamilies. Superfamilies in CATH were mapped to a number of related resources (InterPro, Pfam, SCOP, UniProtKB) as a means to provide evidence for names that were unique and informative while still representing the potentially diverse members in each cluster. This manual curation work resulted in 1,255 superfamily names being reviewed and standardised with respect to equivalent clusters in InterPro. It also generated names and detailed descriptions for an additional 1215 CATH superfamilies that had not yet been annotated.

### Alignment of query sequences against FunFams

**Figure 3.**
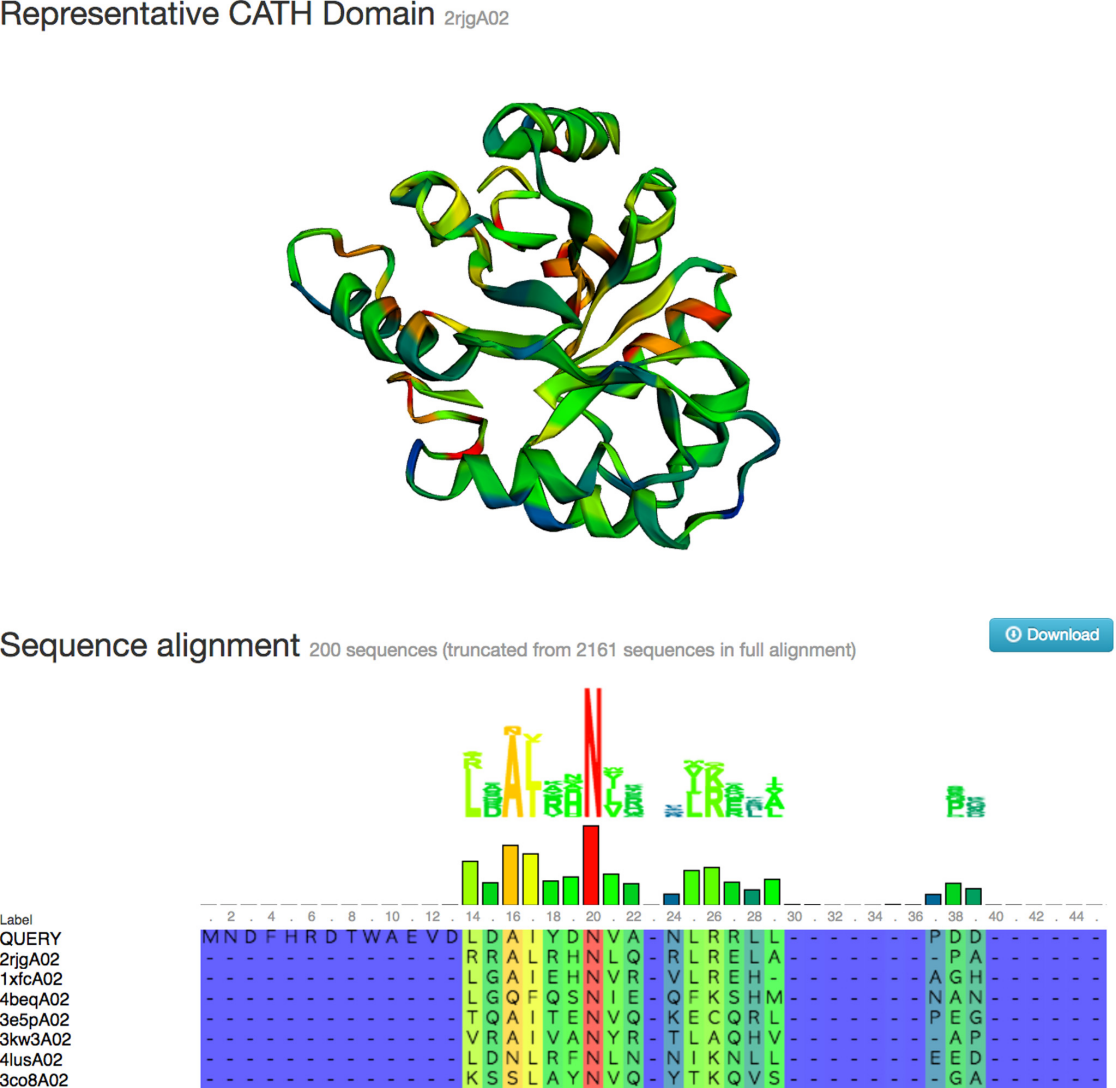
Screenshot showing a query sequence aligned to a matching CATH FunFam (following a sequence search). Sequence conservation is calculated for each position in the alignment (blue is low conservation, red is high conservation) and these colours are mapped to a representative structure (if one is available).

The FunFHMMER server is a web tool that allows users to search a query protein sequence against a library of FunFam HMMs (11). A search typically takes a few seconds and then the user is provided with a list of significant FunFam matches with a visual indication of the location of each match on the original query sequence. In previous releases, the user was able to click through to web pages describing these FunFams in more detail (e.g. to view a multiple sequence alignment with highly conserved positions highlighted on a representative 3D structure), however they were not able to see their own sequence in that alignment. A highly requested feature has been to provide users with the opportunity to view their own query sequence aligned to this structure. This feature has been added and the query sequence is automatically inserted as the first sequence in the FunFam alignment when following links from the search page (Figure 3).

### Visualising structural context of domains

**Figure 4.**
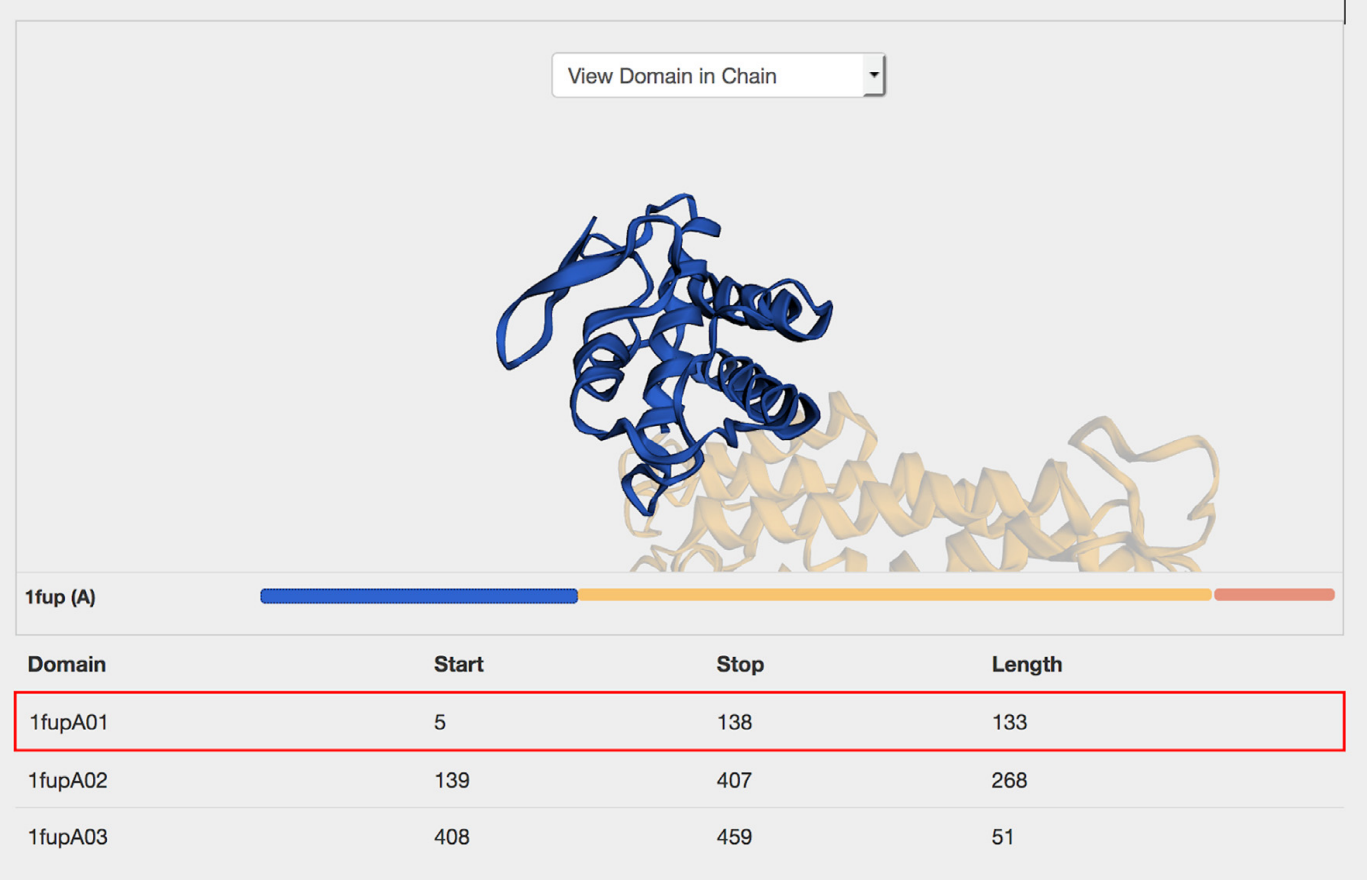
Interactive links between 3D structure (3DMol.js) and multi-domain architecture (MDA) allow the user to view each domain in the context of the full chain.

While it is important to consider CATH domains as semi-independent globular units, it is also important to consider the context in which each domain appears in the native protein, ie the multi-domain architecture. This is crucial when considering functional annotations (such as GO terms) since these are made at the protein level, rather than the domain level. To highlight the structural context of domains within the full protein, a new interactive component has been added to the pages that combines a 3D structural viewer (3DMol.js (24)) with a simple 1D schematic of the multi-domain architecture (MDA) of the protein chain (Figure 4). This enables users to view the domain within the context of the PDB chain (‘View Domain in Chain’) or in the context of the full PDB (‘View Domain in PDB’). In these modes, clicking a domain in the MDA adds focus to the domain in the 3D viewer while ghosting the surrounding domain partners.

## CONCLUSION

This article describes the latest release of CATH version 4.2 that contains over 430,000 structural domains and over 95 million predicted domain sequences. This represents a huge increase in coverage both in terms of coverage of the PDB and of sequences in UniProtKB.

A number of highly requested features have also been implemented in our web pages: allowing the user to view an alignment between their query sequence and a representative FunFam structure and providing tools that make it easier to view the full structural context (multi-domain architecture) of domains and chains.

## DATA AVAILABILITY

Daily snapshot of latest annotations (CATH-B) is available to download at ftp://orengoftp.biochem.ucl.ac.uk/cath/releases/daily-release.

CATH+ data is available to download at ftp://orengoftp.biochem.ucl.ac.uk/cath/releases/all-releases/v4_2_0/ and can be browsed through the web site at http://www.cathdb.info

Datasets of non-redundant domains are available to download at: ftp://orengoftp.biochem.ucl.ac.uk/cath/releases/latest-release/non-redundant-data-sets/
